# American Academy of Dental Science

**Published:** 1891-04

**Authors:** 

**Affiliations:** American Academy of Dental Science


### AMERICAN ACADEMY OF DENTAL SCIENCE.

The American Academy of Dental Science held its regular monthly meeting in the Boston Medical Library Association rooms, October 8, 1890. President Seabury in the chair.

The paper of the evening was read by Dr. Wm. H. Potter, subject, "Creolin."

(For Dr. Potter's paper, see p. 209.)

*Dr. Williams.*—I think the paper by Dr. Potter is an excellent one, showing thorough research and giving a full exposition in brief of the qualities of the drug. I had a little experience with creolin a year or so ago, and in my use of it I found that it had antiseptic qualities to a large extent, but the odor is objectionable.

It is called a deodorizer, but I should think that superodorizer would come nearer to the truth. I ceased to use it on account of its strong tarry odor, which is disagreeable to patients, to some more disagreeable than creosote. I had before that used Little's soluble phenyle, a somewhat similar thing, but the sale of this was discontinued on account of its being an infringement. I have also used the "royal disinfectant," which they say at Metcalf's is the same thing as soluble phenyle. It makes a milky solution in water, and has a less tarry odor than creolin. To me creolin is not unpleasant; its odor indicates a positive purifying, corrective quality, which gives the impression that it will do some good, but to the nasal sense of most people it is rather disagreeable. It has one advantage that the essential oils of course have not,—it can be used in aqueous solution, but the essential oils are more durable; the effects last longer and are not so easily dissipated. The coal-tar preparations do not so readily penetrate the tubuli. If we wish to saturate a dead root, for instance, unless we put a little alcohol with them, they will not be well absorbed. I have generally preferred for such use the essential oils, or a slight amount of alcohol sometimes modified with glycerin. There is also a comparatively new preparation which is called liquid cosmoline, which they tell me at Metcalf's corresponds to glycerin,—that is, its relation to the coal element is the same as that of glycerin to animal fat. It is practically liquid vaseline. A little alcohol will thin it to such an extent that it will be absorbed. Of course, as Dr. Potter says, in cases of fistula or abscess something of the nature of creolin or phenyle is often preferable to the essential oils.

*Dr. Eames.*—I can only add a word in thanks to the author of the paper. There seems to me not much further to be said in regard to this particular agent. My experience, and that of my friends, is that phenyle is now, to quite an extent, used as a substitute for creolin. The use of creolin has, however, been very satisfactory, especially in its kindly action on mucous membranes. I had hoped that some one might bring up pyoktanin, the antiseptic and disinfectant spoken of so highly by medical men.

*Dr. Williams.*—That reminds me that there is an objection to pyoktanin. Although it has apparently very sound claims as a positive antiseptic, it has one disadvantage for us, which is that it gives a blue color,—produces a blue stain. There is a pyoktanin which gives a yellow color, and which perhaps might not be quite as objectionable, but neither of them could be employed for use in the teeth with any degree of satisfaction.

In my use of creolin, or the phenyle solution, both similar preparations, the creolin being the stronger, I have sometimes found a weak watery solution very useful in saturating a cement which was to be put into a deep cavity where a little softened dentine remained in the bottom covering the pulp. I would treat the cavity first with such strictly antiseptic solutions as I thought the tooth would bear, and then cover its deep parts with Wilson's non-irritant (said to be a magnesium cement) or plaster of Paris wet up with a weak watery solution of creolin or phenyle, these drugs maintaining an antiseptic influence in the cavity. Over this layer an oxyphosphate cement, or whatever is best, may be placed. I prefer something of that softer nature in the very depth of the cavity, as the pulp is less apt to be rebellious, and the filling can be graduated with harder materials as you get farther away from the pulp.

*Dr. Andrews.*—Speaking of mixing antiseptics with the different powders for the purpose of covering a pulp which was nearly exposed, calls to my mind a paper which was prepared by Dr. Charles Atkinson, of New York, to be read at Berlin. Unfortunately, it was voted there that no paper could be read whose author was absent, so that among other excellent papers it was thrown aside. Dr. Atkinson sent to me, with the paper, some thirty specimens of oxyphosphate fillings, mixed with different antiseptics. They were all in hard cakes, and most of them seemed quite as hard as they usually are when mixed with nothing but the fluid, while the odor of the different antiseptics used was very perceptible. His paper spoke of the importance of using these medicated cements over exposed pulps, and said that a good deal of the antiseptic material can be mixed up with the powder and yet make a good hard filling.

*President Seabury.*—How are the antiseptics mixed?

*Dr. Andrews.*—I do not know his method. I suppose the antiseptic, such as oil of cassia, or carbolic acid, is mixed with the powder first.

*Dr. Smith.*—In a recent issue of the *Boston Medical and Surgical Journal* some writer cautions the medical fraternity against the use of creolin on the ground that it was liable to cause a humor. I presume Dr. Potter may have seen that. It was in the September number, I think, of the *Medical and Surgical Journal.* Speaking of antiseptics as a whole, after reading Dr. Miller's late paper on this subject, one has to come to the conclusion that the supposed new antiseptics are of but little value as antiseptics, and we have to fall back on such agents as carbolic acid and bichloride of mercury. According to his experiments, iodoform, which the medical fraternity have hitherto relied on, amounts to but little as a positive antiseptic. Perhaps when creolin receives his attention it will be classed in the same list.

*Dr. Williams.*—I think Dr. Miller speaks more particularly of the active agents, which are called germicides or bacteriacides, instead of those that have simply an aseptic influence,—he speaks more of those that will actually kill the bacteria. I think it is now claimed that iodoform is not so active, but very good to keep up the resistance, as in preserving the tissue, or helping it to resist the advance of germ-life.

*Dr. Andrews.*—Dr. Black, as a result of his experiments, stated, if I remember rightly, that germs not only lived in iodoform, but they increased in it.

*Dr. Eames.*—There is so much ambiguity concerning the meaning of the words "antiseptic" and "disinfectant" that I would like to know the opinions of the members with regard to it. One authority says that an antiseptic prevents the formation of germs and a disinfectant kills them. Authorities conflict in their definition of these terms. I am led to conclude that both are antizymotics, having the power to arrest fermentative processes. Antiseptics prevent or retard septic decomposition, disinfectants destroy the specific germs of disease, many of which are due to the action of microbes. Therefore many antiseptics are also disinfectants.

*Dr. Potter.*—The terms disinfectant, antiseptic, and germicide are often used as synonymes. According to Edes's "Therapeutics and Materia Medica," p. 245, however, the following distinctions should be made: Antiseptics are such substances as prevent putrefaction or septic decomposition, while disinfectants are such substances as render organic matter not only incapable of putrefaction itself, but of becoming the starting-point either of putrefaction or any of the morbid processes dependent on peculiar organized ferments when brought in contact with other organic matter. Germicide is a term which in the present status of the germ-theory is nearly or quite equivalent to disinfectant.

William H. Potter, D.M.D.,
*Editor American Academy of Dental Science.*

